# Benefits of INSTI-Based Regimens in a Real-World Setting of People Living With HIV-1 in Colombia

**DOI:** 10.1155/ijm/9081023

**Published:** 2025-09-10

**Authors:** Juan C. Alzate-Angel, John D. Loaiza, Federico Perdomo-Celis, Juan C. Hernandez, Ximena Galindo-Orrego, Mónica Mantilla-Suárez, Fanny Guzman, Juan Carlos Cataño, Francisco J. Diaz, María T. Rugeles, Natalia A. Taborda

**Affiliations:** ^1^Grupo Micología Médica y Experimental, Corporación para Investigaciones Biológicas, Medellín, Colombia; ^2^Grupo Inmunovirología, Facultad de Medicina, Universidad de Antioquia UdeA, Medellín, Colombia; ^3^Instituto de Genética Humana, Pontificia Universidad Javeriana Facultad de Medicina, Bogotá, Colombia; ^4^Infettare, Universidad Cooperativa de Colombia Facultad de Medicina, Medellín, Colombia; ^5^Corporación de Lucha Contra el SIDA, Cali, Colombia; ^6^Virrey Solis IPS, Bogotá, Colombia; ^7^Núcleo Biotecnología Curauma, Pontificia Universidad Católica de Valparaíso, Valparaíso, Chile; ^8^Infectious Diseases Section, Facultad de Medicina, Universidad de Antioquia UdeA, Medellín, Colombia; ^9^Grupo de Investigaciones Biomédicas Uniremington, Corporación Universitaria Remington Facultad de Ciencias de La Salud, Medellín, Colombia

**Keywords:** first-line antiretroviral therapy, integrase strand transfer inhibitors, nucleoside (tide) reverse transcriptase inhibitors, protease inhibitors, viral suppression

## Abstract

**Objective:** The objective is to compare the clinical, virological, and immunological characteristics of people living with HIV-1 who were treated with first-line integrase inhibitors (INSTIs) versus those treated with other antiretrovirals.

**Methods:** This is a descriptive observational, retrospective, two-center study. We selected individuals who began antiretroviral therapy (ART) within the first year of diagnosis and who had received at least 1 year of treatment. Their therapy consisted of two nucleoside/nucleotide reverse transcriptase inhibitors, along with a third medication (INSTI, nonnucleoside reverse transcriptase inhibitor (NNRTI), or protease inhibitor). The primary comparison was between individuals who initiated ART containing INSTIs and those who initiated ART containing NNRTIs or protease inhibitors. We evaluated clinical outcomes, residual viral load, circulating proviral DNA, inflammation, and cardiovascular risk markers, T cell count and phenotype, and the frequency of HIV-specific T cells.

**Results:** Individuals who received INSTI-based regimens started treatment at a more advanced disease stage and had a significantly shorter ART duration at study inclusion than individuals who did not receive INSTI-based ART. Individuals receiving INSTIs achieved CD4^+^ T cell counts at levels comparable to those within the non-INSTI group, as well as similar levels of virological, immunological, and cardiovascular markers. Nonetheless, those in the INSTI-based group had higher CD8^+^ T cell counts and lower CD4/CD8 ratios, as well as lower high-density lipoprotein levels.

**Conclusions:** INSTI-based regimens offer benefits to Colombian people living with HIV-1, who are starting ART, including improved and faster virological and CD4^+^ T cell reconstitution. This occurs despite treatment initiation for advanced disease.

## 1. Introduction

Integrase strand transfer inhibitors (INSTIs) are among the newest classes of antiretroviral drugs used for the standard treatment of human immunodeficiency virus type one (HIV-1) infection. Clinical trials that compared first-line antiretroviral therapy (ART) regimens that include this class of drugs versus those that contain a nonnucleoside reverse transcriptase inhibitor (NNRTI) or a protease inhibitor demonstrated that INSTIs offer high tolerability and efficacy, as well as a faster time to virologic suppression [1–4]. Second-generation INSTIs, such as dolutegravir (DTG) and bictegravir (BIC), have a high genetic barrier, which makes them helpful in treating multidrug-resistant HIV-1 [5]. Moreover, INSTIs exhibit less severe side effects and, in addition to viral suppression, may contribute to reducing the reservoir size and the level of immune activation [6–10]. For these reasons, INSTIs are currently recommended for first-line ART regimens for HIV-1 infection [11].

The choice of INSTIs in first-line regimens depends on several patient conditions, such as drug tolerability, toxicity, and coexisting medical and social conditions [12, 13]. However, despite the efficacy demonstrated in clinical trials, INSTI-based therapy in low-resource settings may depend on external factors, particularly those related to high drug costs and accessibility [14]. Therefore, real-world comparisons of first-line ART regimens may differ from those observed in clinical trials [15]. INSTIs were recently introduced as first-line ART regimens in Colombia, a low-resource country [16]. However, given the limitations in drug accessibility, currently, the use of INSTIs in first-line ART regimens is mainly restricted to individuals who are diagnosed at an advanced disease stage.

The limited availability of INSTIs and the high cost of these medications present significant obstacles to HIV treatment, particularly in low- and middle-income countries. The steep price of drugs like long-acting cabotegravir may reduce the number of individuals who can receive treatment within fixed prevention budgets, hindering efforts to lower HIV incidence [17]. Furthermore, the transition to new antiretroviral therapies in resource-limited settings is complicated by factors such as the lack of clinical data on key populations and restricted access to viral load and genotypic resistance testing, making the implementation of more effective treatments increasingly challenging [18]. Thus, here we aimed to evaluate clinical, virological, immunological, and cardiovascular parameters in Colombian people living with HIV-1 who were treated with first-line integrase inhibitors versus other antiretrovirals. The study period encompasses the introduction of INSTIs as first-line treatment in the Colombian guidelines. It reflects the specific conditions under which these drugs are used in people living with HIV-1.

## 2. Methods

A descriptive, exploratory study was conducted based on the analysis of two groups of patients categorized according to their exposure to the third class of the antiretroviral drug. The clinical, virological, and immunological status at the initiation of ART (assessed retrospectively) was compared with the virological and immunological status at the time of study entry (assessed cross-sectionally).

### 2.1. Patient Selection and Data Collection

All procedures were conducted by the principles of the Declaration of Helsinki and Colombian Resolution 8430 of 1993. The ethics committee considered the present work a minimum-risk investigation. All individuals provided informed consent, which was previously approved by the Research Bioethics Committee CBE-SIU, Universidad de Antioquia (Code 19-08-844). Patients were asked for a copy of their medical history and test results from diagnosis to recruitment. The confidentiality and anonymity of each individual were maintained. All data obtained were subject to confidentiality using a unique identification number for each individual. Only personnel in charge of the study had access to the database. In addition, the recommendations of the guidelines for implementing the patient safety policy and the guide of good practices for patient safety in health care were followed. The databases of two HIV-1 healthcare institutions in Medellín (IPS Virrey Solís) and Cali (CORPOSIDA), Colombia, were reviewed from January 2013 to February 2022. Participants were consecutively selected if they met the following inclusion criteria: (i) were over 18 years of age and had a confirmed diagnosis of HIV-1 infection according to Colombian guidelines [19], (ii) had initiated ART within the first year of diagnosis and had been on treatment for at least 1 year at the time of recruitment, and (iii) had initiated ART with a regimen based on two nucleoside/nucleotide reverse transcriptase inhibitors plus a third medication, which could be an INSTI, a NNRTI, or a protease inhibitor. Clinical records and laboratory results were obtained from each participant's medical history at the time of treatment initiation. Additionally, the evolution of CD4^+^ T cell counts and viral loads was retrospectively documented up to the point of study entry.

The primary comparison was between individuals who initiated ART with INSTI as the third medication and those who initiated ART with NNRTIs or protease inhibitors as the third drug in first-line regimens. Considering previous studies that showed improved virological and immunological reconstitution with the use of INSTIs in various ART regimens [6–9], the study sample size was designed to detect differences in viral suppression between the two groups of participants, 12 months after initiating ART, with a moderate effect size (0.6), which is considered reasonable given that the study is aimed at detecting a 10%–15% difference in the proportion of virological suppression, with a Type I error probability of 5% and statistical power of 80%. The final study sample included 72 participants. Under these conditions, the minimum detectable difference (*δ*) between groups increases to 20%, assuming the same power and significance level. Using the standard formula for comparing two independent proportions: *δ* = √(2(*Z*_1−(*α*/2)_ + *Z*_1−*β*_)^2^.*p*(1 − *p*))/*n*.

### 2.2. Laboratory Procedures

At the time of inclusion in the study, a venous blood sample was taken from each participant to obtain whole blood, plasma, serum, and peripheral blood mononuclear cells (PBMCs) for the evaluation of virological, immunological, and cardiovascular parameters, as described below. Whole blood was used to obtain a complete automated blood cell count and to evaluate the T cell phenotype. Finally, PBMCs were used to quantify the amount of circulating proviral DNA and to evaluate the frequency of HIV-specific T cells.

#### 2.2.1. Evaluation of Soluble Inflammation and Cardiovascular Risk Markers

The levels of sCD14 were determined in plasma using the human sCD14 ELISA Kit (MyBioSource, Cat.: MBS2514176). The levels of high-density (HDL) and low-density (LDL) lipoproteins, triglycerides, and total cholesterol were determined in serum by a colorimetric assay, while the plasma levels of CRP and D-dimer were determined by immunoturbidimetry and immunochromatography, respectively.

#### 2.2.2. Residual Viral Load Determination

The residual viral load in plasma at a single RNA copy/milliliter level was determined using digital PCR (dPCR) with the QIAcuity digital PCR thermocycler. Viral RNA was extracted from 1 mL of plasma using the commercial Quick-RNA Viral Kit (Cat: R1035-E, ZYMO), after centrifugation of the sample at 20,000×*g* for 90 min and removal of 800 *μ*L of the supernatant. cDNA synthesis was performed with the commercial iScript cDNA Synthesis Kit (Cat: 1708891, Bio-Rad), following the manufacturer's instructions. Once cDNA was obtained, digital PCR assembly was carried out using the QIAcuity Probe PCR kit (Qiagen, 10 *μ*L), with the primers 6F (CATGTTTTCAGCATTATCAGAAGGA) and 84R (TGCTTGATGTCCCCCCAC) (both amplifying a conserved region of the *gag* gene, at 0.8 *μ*M each), and the Gag. P FAM-CCACCCCACAAGATTTAAACACCATGCTAA-BHQ1 probe (at 0.3 *μ*M). The sample volume was 15 *μ*L, resulting in a final reaction volume of 40 *μ*L. Samples were analyzed in duplicate on 24-well plates, each with 26,000 partitions, using the following PCR protocol: initial temperature of 95°C for 5 min, followed by 50 cycles of 95°C for 15 s and 60°C for 1 min. The plate was read in the SYBR/FAM channel. Prior to this determination, a two-phase standardization was performed, first using real-time PCR and subsequently dPCR (Figure S1). For absolute quantification of residual viral load, standard curves were generated using the plasmid pfNL43-dE-EGFP, which contains the whole genome of HIV-1.

### 2.3. *Ex Vivo* Phenotype of Circulating T Cells

The differentiation and activation phenotype of circulating T cells was determined by multiparametric flow cytometry. For this, 100 *μ*L of whole blood was incubated for 30 min with optimized doses of fluorochrome-conjugated antibodies (Table S1). Subsequently, red blood cells were lysed with 2 mL of FACS Lysing 1X solution (BD) for 25 min. Finally, cells were washed with 1% fetal bovine serum (FBS) in 1X PBS (FACS buffer) and resuspended in 2% paraformaldehyde for acquisition on a LSRFortessa flow cytometer (BD). T cell absolute numbers were calculated according to the absolute white blood cell count. The representative gating strategy is shown in Figure S2.

#### 2.3.1. PBMC Isolation

A Ficoll density gradient (Ficoll Paque, GE Healthcare) was used to isolate PBMCs, followed by washing with RPMI 1640 medium supplemented with 10% FBS and 100 U/mL penicillin, 100 mg/mL streptomycin, and 2 mM L-glutamine (complete medium). Subsequently, the PBMCs were counted and resuspended in FBS containing 10% DMSO and then cryopreserved in liquid nitrogen. All experiments were conducted using cryopreserved PBMCs.

#### 2.3.2. Quantification of Proviral DNA in Total PBMCs

DNA was extracted from 5 × 10^6^ PBMCs using the Quick-DNA Miniprep Plus Kit (Cat: D4068, ZYMO). Eight microliters of the DNA was used in the presence of the following primers and probes: primers directed at HIV-1 (conserved region of the LTR sequence): NEC005-F primer sequence: GCC TCA ATA AAG CTT GCC. NEC131-R primer sequence: GGC GCC ACT GCT AGA GAT TTT. MGB-LTR probe sequence: /56-FAM/AA GTR GTG TGT GCC C/3MGBEc. Probe directed at CD4: TaqMan Gene Expression Assay, VIC primer-limited (Applied Biosystems, Ref: 4448485). The probes and primers directed at HIV-1 were used at 3 and 8 *μ*M, respectively. The master mix used for the PCR reaction was Luna Universal Probe qPCR Master Mix (10 *μ*L, New England Biolabs, Cat. M3004S). The final volume per PCR reaction was 20 *μ*L, and the following PCR protocol was used: initial temperature of 95°C for 5 min, followed by 45 cycles of 95°C for 20 s and 60°C for 45 s. The plate was read in the FAM channel (HIV-1 probe) and the VIC channel (CD4 probe). A CFX96 thermocycler was used. The relative level of viral reservoirs was determined using the delta-Ct formula, with the CD4 gene serving as a reference for a constitutive gene. Values were log-transformed.

#### 2.3.3. Evaluation of the Frequency of HIV-Specific T Cells

The AIM assay was used to determine the frequency of HIV-specific T cells [1]. Total PBMCs were thawed and left to rest for 12 h. Then, cells were cultured in 96-well V-bottom polystyrene culture plates (Corning) in a total volume of 200 *μ*L, at 2 × 10^6^ cells/mL, under the following conditions: (i) negative control: anti-CD28 and CD49d antibodies (both at 1 *μ*g/mL); (ii) anti-CD28 and CD49d antibodies plus a pool of consensus HIV-1 Gag peptides; (iii) positive control: PMA/ionomycin (50 and 500 ng/mL, respectively, Sigma). All conditions were incubated for 12 h at 37°C, 5% CO_2_, in the presence of IL-2 and DNase I (both at 10 U/mL). After culture, cells were washed with 1X PBS, stained with the Fixable Viability Dye eFluor 506 (Thermo Fisher), and then incubated for 30 min with optimized doses of fluorochrome-conjugated antibodies (Table S2). Finally, cells were washed with FACS buffer and acquired on a LSRFortessa flow cytometer (BD). A representative gating strategy is shown in Figure S3.

### 2.4. Statistical Analysis

GraphPad Prism v.10.0 (GraphPad Software) and Jamovi v.2.5.1 (https://www.jamovi.org/) were used for the statistical analyses. The data are presented as medians and interquartile ranges (IQRs). The Mann–Whitney *U* test was used for the univariable comparison of two unpaired datasets. Categorical variables are reported as counts and percentages and were compared with Fisher's exact test. All values of *p* < 0.05 were considered statistically significant.

## 3. Results

### 3.1. Baseline Characteristics of the Study Cohort

In this study, we included 37 individuals who started first-line INSTI-based ART regimens and 35 who started NNRTI- or protease inhibitor–based regimens (here referred to as non-INSTI–based regimens) (Table 1). At the time of ART initiation, in the INSTI-based group, 22 (59.5%) individuals received DTG, 8 (21.6%) received raltegravir (RAL), and 7 (18.9%) received elvitegravir/cobicistat (EVG/c). In the non-INSTI–based group, 29 (82.9%) individuals received efavirenz (EFV), 4 (11.4%) received protease inhibitor (two received atazanavir/ritonavir [ATV/r], one darunavir/ritonavir [DRV/r], and one lopinavir/ritonavir [LPV/r]), and 2 (5.7%) received nevirapine. Regarding the ART backbone received by all study participants, 39 (54.2%) individuals received a combination of tenofovir disoproxil fumarate/emtricitabine (TDF/FTC), 20 (27.8%) received abacavir/lamivudine (ABC/3TC), 7 (9.7%) received tenofovir alafenamide/emtricitabine (TAF/FTC), and 5 (6.9%) received zidovudine/lamivudine (AZT/3TC). Only one individual started ART with a dual regimen based on DTG/3TC.

Individuals who initiated first-line INSTI-based regimens had a significantly shorter ART duration at study inclusion compared to those who did not receive INSTI-based ART (Table 1). In addition, a higher percentage of individuals in the INSTI-based group than in the non-INSTI–based first-line ART group were diagnosed at a more advanced disease stage (20.0% vs. 37.8% in Stage 3, respectively, Table 1).

### 3.2. Longitudinal Follow-Up of Virological and CD4^+^ T Cell Reconstitution After Therapy

**Figure 1:**
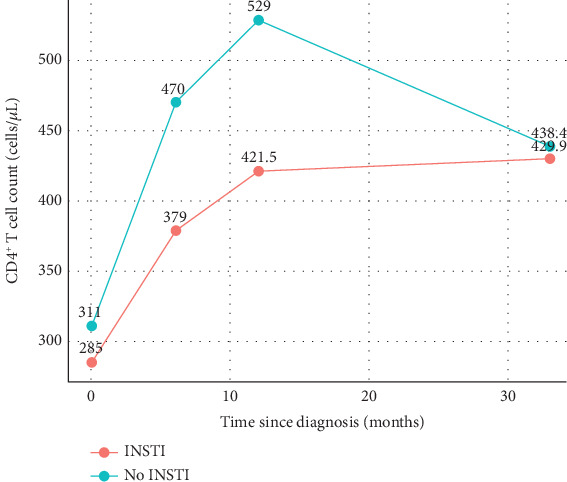
Longitudinal CD4^+^ T cell counts. Median CD4^+^ T cell counts were evaluated since HIV-1 diagnosis in individuals receiving INSTI-based or non-INSTI–based ART regimens. The median is shown.

We longitudinally evaluated the blood viral load and CD4^+^ T cell count at 0, 6, 12, 24, and 36 months after HIV-1 diagnosis, as well as at the time of study inclusion. Considering that both groups of individuals started ART at a median of 1 month after diagnosis (Table 1), these time points also reflect the level of viral suppression and CD4^+^ T cell reconstitution after therapy. At 6 months, more than 80% of individuals in both groups reached viral suppression (Table 1). Although not statistically significant for making inferences to the general population, in the evaluated sample, the proportion of individuals with virological suppression at 12 months was higher among those who initiated treatment with an INSTI-based regimen (Table 1). Finally, at the time of study inclusion, both groups had comparable percentages of viral suppression (Table 1). On the other hand, we observed a longitudinal increase in CD4^+^ T cell counts in both study groups (Figure 1), although those who started first-line INSTI-based treatment tended to have lower counts than those in the non-INSTI–based group at 0, 6, and 12 months (Table 1 and Figure 1). This is interesting considering that those who started first-line INSTIs had been on ART for a significantly shorter duration when the study began (Table 1 and Figure 1). We also evaluated the rate of ART regimen change in both study groups before study inclusion. This percentage tended to be greater in the group that started an ART regimen without INSTIs, and the drug change was generally due to poor drug tolerance (five patients), drug resistance (one patient), or tuberculosis coinfection (one patient). Notably, 3 out of 11 individuals who needed a change in their first-line ART regimen were subsequently treated with INSTIs for at least 23 months before study inclusion. The changes in these patients were EFV for the RAL, DRV/r for the DTG, and ATV for the RAL. Since these three individuals received INSTIs for a duration comparable to that of other individuals in the INSTI group (median of 23.7 months [Table 1]), we included them in this group for further analysis.

### 3.3. Virological, Immunological, and Cardiovascular Characteristics of People Receiving ART Regimens Containing INSTIs Versus Other Antiretrovirals

We next analyzed several virological, immunological, and cardiovascular markers in the participants. We did not observe significant differences in the residual viral load or circulating proviral DNA between the two groups of individuals (Table 2). However, we observed lower percentages of CD4^+^ T cells, as well as higher percentages and absolute counts of CD8^+^ T cells, along with a lower CD4^+^/CD8^+^ T cell ratio, in the INSTI-based group than in the non-INSTI–based group (Table 2). We also evaluated the inflammatory markers CRP and sCD14 and observed no differences between the study groups (Table 2). Similarly, we did not observe differences in the frequencies of CD4^+^ and CD8^+^ T cell memory subpopulations (identified based on the expression of CCR7 and CD45RA, Table 2) or in the frequency of activated HLA-DR^+^ CD38^+^ T cells (Table 2). Moreover, we did not find differences in the frequency of circulating HIV-specific T cells (measured upon ex vivo Gag peptide stimulation, Table 2) or in the proportion of cells responding to polyclonal stimulation (as an indicator of global T cell functionality, Table 2). Finally, we evaluated the levels of total cholesterol, LDL, HDL, triglycerides, and D-dimer, which are commonly used to assess cardiovascular risk in people living with HIV-1. Overall, the participants in both study groups exhibited normal levels of these markers, although individuals receiving INSTIs had significantly lower levels of HDL than those who received other drugs (Table 2).

## 4. Discussion

The use of INSTIs in first-line ART regimens has shown benefits compared to regimens that do not include them, such as improved viral suppression and immune reconstitution [9, 20]. However, in Colombia, these medications are not widely used and are not a priority in real-world clinical scenarios. As a result, there is not enough data on the effect of these medications in first-line ART regimens in people living with HIV-1 in this country. Therefore, in this study, according to our knowledge, for the first time, we evaluated the clinical, virological, immunological, and cardiovascular markers of first-line INSTI-based ART regimens in Colombian people living with HIV-1. Our findings indicate that despite INSTIs being reserved for late-stage disease conditions, patients receiving these drugs rapidly reach clinical, virological, immunological, and cardiovascular features comparable to those receiving other ART regimens for a longer time.

The duration of ART is a factor that determines immune reconstitution [21–23]. Here, we show that individuals receiving INSTIs rapidly increased CD4^+^ T cell counts to levels comparable to those who received other drugs. These data align with previous studies showing that INSTI-based regimens result in a more rapid virological suppression among treatment-naïve individuals than NNRTI- or protease inhibitor–based regimens in comparable real-world clinical settings [24]. Similarly, previous studies have shown that ART regimens containing RAL (both first-line and intensification) lead not only to an increase in the CD4^+^ T cell count but also to the reconstitution of naïve and central memory populations [25, 26], which are markers of early immune reconstitution in treatment-naïve people living with HIV-1 [27]. A mechanism likely explaining this process is the perturbation in the viral reservoir and the decrease in residual viral replication, with the consequent reduction of immune activation by INSTIs [7]. Consistently, we observed that individuals who started INSTI-based regimens achieved levels of circulating proviral DNA, residual viral load, CRP, and sCD14, as well as frequencies of activated T cells, comparable to those who started non-INSTI–based therapy, even though the latter had a twofold longer duration of ART. Similar observations have been made for treatment-naïve individuals with first-line EVG/c/FTC/TDF, who exhibited greater decreases in CRP and sCD14 than those who received EFV/FTC/TDF [28].

Another finding in our study was that individuals who received INSTI-based regimens had greater CD8^+^ T cell counts and lower CD4^+^/CD8^+^ T cell ratios than individuals who did not receive INSTI-based regimens. These results are in line with the existing evidence that not all immunological parameters are reconstituted equally upon ART. Indeed, we previously showed that the CD8^+^ T cell count and CD4^+^/CD8^+^ T cell ratio, concomitant with altered effector functions, improved only slightly after short-term ART (< 25 months) [29–31]. Some studies have suggested that these two parameters are associated with an increased risk of non-AIDS events, explained by increased immune activation and the presence of T cells exhibiting an immunosenescent phenotype [32]. However, considering that previous studies have shown improved CD4/CD8 ratio normalization with first-line INSTI-containing ART compared with other regimens [33–35], we speculate that a longer duration of INSTI-based ART in our study cohort would be required to reconstitute these parameters. In addition, it is important to consider that three patients from the total INSTI-based group were previously treated with other antiretrovirals. Although it could affect these observations, as previously reported [36], clinical characteristics and treatment duration with INSTI in these patients were similar to the rest of the INSTI-based group patients, mitigating the impact on the intergroup balance.

Other variables, such as cytomegalovirus infection (not evaluated here), may influence the CD4/CD8 ratio reconstitution level in these individuals [37]. This ratio can be affected by coinfection or inflammation, as shown in a study where patients with hepatitis C virus coinfection and cardiovascular disease exhibit lower CD4/CD8 ratios, associated with the duration of the HIV infection, nadir CD4^+^ cell count, and AIDS [38]. In addition, microbiota composition could be related to immune reconstitution. Some studies have shown that patients starting HIV treatment with INSTI exhibit recovery of gut microbiota and its metabolites, as well as decreased levels of proinflammatory cytokines such as IP-10, with an improved gut ecosystem in those with CD4/CD8 ratio > 1, classified as immunological responders [36]. Nonetheless, although a lower normalization rate, it should be noted that in most individuals from our two study groups, the CD4^+^/CD8^+^ T cell ratio was greater than 0.5, and values above these levels have been associated with a decreased risk of non-AIDS–related events [39, 40].

We also evaluated the ability of CD4^+^ and CD8^+^ T cells to respond to antigen-specific and polyclonal stimulation in vitro. These assays help reveal potential dysfunction that these cells may have due to chronic stimulation and altered TCR signaling [41, 42]. We showed that although individuals who started INSTI-based ART had more severe immunodeficiency, their cells could respond to these stimuli at levels comparable to those of individuals who received other ART regimens. However, we did not evaluate the functional properties of Gag-specific T cells (such as cytokine production, proliferation, and expression of cytolytic molecules), which may be negatively impacted by several INSTIs [43, 44]. The clinical consequences of these effects are unknown. Additional studies are necessary to elucidate the impact of INSTIs on additional components of the immune system and immunological reconstitution.

Cardiovascular diseases constitute one of the main comorbidities in people living with HIV-1 in the era of ART, and substantial efforts are ongoing to develop novel strategies to reduce cardiovascular risk and to identify ART-related toxicity [45]. A recent work showed that first-line INSTI-based ART is not associated with an increased risk for cardiovascular events [46].

However, in the RESPOND cohort, INSTI initiation was associated with increased cardiovascular risk within the first 24 months, although thereafter, it decreased to levels similar to those of individuals who were not exposed to these drugs [47]. In addition, the analysis of the incidence of dyslipidemia in the same cohort showed that participants taking INSTIs had a lower incidence of this condition than did those receiving protease inhibitors but a higher incidence than did those receiving NNRTIs [48]. Nonetheless, other studies have shown that although there is an increase in the mean concentrations of total cholesterol and LDL with the use of second-generation DTG, it has a safer lipid profile in comparison with ART schemes containing NNRTIs, IP, or first-generation RAL [49]. Thus, the effect of INSTIs may differ depending on population characteristics, as well as the type of INSTI used. In our study, comparable levels of total cholesterol, LDL, triglycerides, and D-dimer were found between the INSTI-based group and the non-INSTI–based group, suggesting a lack of effect of INSTIs on the incidence of dyslipidemia in these real-world settings. However, lower levels of HDL were found in individuals receiving INSTI-based ART. Low HDL is an important risk factor for cardiovascular disease [50] and is one of the most common dyslipidemia abnormalities in people living with HIV-1 [51]. In Colombia, more than 80% of people living with HIV-1 have dyslipidemia, along with traditional risk factors for coronary artery disease, such as diabetes and obesity [52]. Thus, the lower levels of HDL in the INSTI group highlight the need for strict monitoring of cardiovascular risk markers. Additional long-term longitudinal studies evaluating the impact of INSTIs on Colombian people living with HIV-1 are needed.

Our study has limitations. First, we did not longitudinally evaluate the clinical features or biomarkers in our cohort of individuals. Thus, the long-term effects of INSTI therapy and its potential side effects, such as weight gain, still require further evaluation. Specifically, DTG and BIC have been linked to a significant increase in body weight, particularly among women, black individuals, and adults over 60 years [53–55]. Additionally, DTG and elvitegravir may contribute to neuropsychiatric effects, such as anxiety and stress, with a higher discontinuation rate observed in women and older adults [56–58]. These side effects must be considered in a more complete study, where all these variables can be evaluated to correlate better biological and clinical parameters. In addition, the low number of individuals did not allow for the performance of additional comparisons, such as among different ART schemes or according to age and sex. Our study should be understood as a descriptive, exploratory analysis, in which uncontrolled confounding effects remain possible. These were not addressed due to the study design, which did not include a predefined causal framework. Any attempt to control for confounding in this context could result in a purely statistical exercise lacking the necessary biological plausibility for meaningful interpretation. Given this and the absence of randomization in sampling, the results and observed differences according to the antiretroviral classes analyzed should be interpreted as hypothesis-generating. They may suggest potential associations and underlying mechanisms but cannot be used to establish causal relationships within the scope of the present study. Finally, novel virological and immunological studies in these types of cohorts should evaluate reconstitution in peripheral tissues, which are major sites of HIV-1 replication.

In conclusion, we report for the first time that INSTI-based regimens are safe and offer benefits to people living with HIV-1 who are starting ART in real-world clinical settings, in the context of a developing country where more than 35% of incident cases are diagnosed in the SIDA phase [59]. These benefits include faster virological and CD4^+^ T cell reconstitution, even if treatment is initiated at an advanced disease stage, along with adequate cardiovascular and inflammation marker levels. However, additional studies with extended follow-up are needed.

## Figures and Tables

**Table 1:** Baseline characteristics of the study cohort.

**Parameter**	**INSTI-based (** **n** = 37**)**	**Non-INSTI–based (** **n** = 35**)**	**p** ** value**	**95% CI of difference**
% (*n*) males	72.3 (27)	71.4 (25)	0.88^d^	−18.6, 21.7
Age, years^a^	37.2 (30.6–56.9)	37.8 (29.3–48.4)	0.58^e^	−4.98, 8.42
Time from diagnosis to ART initiation, months^a^	1.0 (0.7–3.0)	1.6 (0.8–4.0)	0.32^e^	−1.00, 0.30
ART duration at study inclusion, months^a^	23.7 (17.2–33.4)	55.9 (34.8–85.4)	< 0.01^e^	−45.57, −18.53
% (*n*) disease stage at diagnosis^b^				Not calculated
Stage 1	18.9 (7)	20 (7)	0.23^d^	
Stage 2	43.2 (16)	60 (21)		
Stage 3	37.8 (14)	20 (7)		
% (*n*) AIDS-associated events	35.1 (13)	22.9 (8)	0.25^d^	−8.7, 31.7
CD4^+^ T cell count at ART initiation, cells/*μ*L^a^	285 (69.4–444.1)	311 (235–477.0)	0.25^e^	(−189.00, 36.00)
Viral load at ART initiation, log^a^	5.1 (3.9–5.7)	4.5 (3.9–5.2)	0.14^e^	−0.13, 0.87
% (*n*/total available data) viral suppression at 6 months after ART initiation^c^	80.6 (29/36)	83.9 (26/31)	0.72^d^	−21.3, 15.8
% (*n*/total available data) viral suppression at 12 months after ART initiation^c^	94.4% (34/36)	79.4% (27/34)	0.06^d^	−1.2, 31.7
% (*n*) viral suppression at study inclusion^c^	89.2 (33)	88.6 (31)	0.93^d^	−14.9, 16.5
% (*n*) ART regime change	21.6 (8)	31.4 (11)	0.35^d^	−29.3, 10.4
% (*n*) ART regime change to INSTI-based	NA	8.6 (3)	0.07^d^	Not calculated

^a^The median (IQR) is shown.

^b^CDC classification 2008.

^c^Viral suppression: < 50 HIV-1 RNA copies/milliliter.

^d^Fisher's test.

^e^Mann–Whitney test.

**Table 2:** Virological, immunological, and cardiovascular characteristics of the study cohort at inclusion.

**Parameter ** ^**a**^	**INSTI-based (** **n** = 40**)**	**Non-INSTI–based (** **n** = 32**)**	**p** ** value ** ^**b**^
Residual viral load, copies/mL	15.3 (5.1–24.4)	10.2 (5.1–18.1)	0.43
Proviral DNA relative levels, log	3 (2.82–3.42)	3 (2.86–3.33)	0.69
% of blood CD4^+^ T cells	34.5 (24.9–47.3)	48.6 (32.2–53.1)	0.05
% of blood CD8^+^ T cells	56.2 (45.3–64.8)	44.2 (37.8–57.8)	**0.02**
CD4^+^ T cell count, cells/*μ*L	435.2 (276.0–640.3)	423.1 (286.9–588.5)	0.58
CD8^+^ T cell count, cells/*μ*L	678.4 (416.4–958.8)	481.1 (290.2–717.8)	**0.01**
CD4/CD8 ratio	0.6 (0.4–1.0)	1.1 (0.6–1.3)	**0.03**
C-reactive protein, mg/L	3.5 (2.0–5.4)	3.1 (1.1–10.9)	0.94
Soluble CD14, ng/mL	16.5 (13.6–18.3)	17.6 (14.1–20.7)	0.09
% CCR7^+^ CD45RA^+^ naïve CD4^+^ T cells	6.0 (2.4–28.4)	22.8 (6.9–27.4)	0.10
% CCR7^+^ CD45RA^−^ central memory CD4^+^ T cells	20.3 (11.9–29.6)	18.4 (11.2–27.9)	0.81
% CCR7^−^ CD45RA^−^ effector memory CD4^+^ T cells	59.8 (35.2–78.1)	47.9 (35.5–74.0)	0.65
% CCR7^−^ CD45RA^+^ effector CD4^+^ T cells	2.8 (1.1–6.3)	2.7 (1.5–7.0)	0.75
% HLA-DR^+^ CD38^+^ activated CD4^+^ T cells	0.9 (0.4–1.8)	1.0 (0.6–1.7)	0.74
% CCR7^+^ CD45RA^+^ naïve CD8^+^ T cells	10.9 (5.1–18.4)	12.7 (6.7–25.4)	0.25
% CCR7^+^ CD45RA^−^ central memory CD8^+^ T cells	4.9 (1.9–9.3)	2.3 (1.4–4.5)	0.07
% CCR7^−^ CD45RA^−^ effector memory CD8^+^ T cells	60.9 (39.2–71.7)	52.7 (41.1–66.6)	0.45
% CCR7^−^ CD45RA^+^ effector CD8^+^ T cells	17.2 (11.6–31.9)	27.5 (18.6–32.9)	0.06
% HLA-DR^+^ CD38^+^ activated CD8^+^ T cells	1.7 (0.7–3.1)	2.1 (1.6–5.1)	0.08
% of CD137^+^ OX40^+^ HIV-specific CD4^+^ T cells	0.01 (0.001–0.052)	0.008 (0.001–0.083)	0.71
% of CD137^+^ CD69^+^ HIV-specific CD8^+^ T cells	0.033 (0.001–0.115)	0.051 (0.001–0.173)	0.67
% of CD137^+^ OX40^+^ polyclonal CD4^+^ T cells	15.7 (7.4–69.6)	6.9 (2–20.4)	0.09
% of CD137^+^ CD69^+^ polyclonal CD8^+^ T cells	17.9 (5.2–30)	7 (5–17.9)	0.18
Total cholesterol, mg/dL	170.5 (160.8–197.8)	192.0 (166.8–207.1)	0.09
Low-density lipoproteins, mg/dL	95.9 (75.7–107.3)	101.9 (87.9–124.3)	0.31
High-density lipoproteins, mg/dL	38.5 (34.2–47)	45 (38.2–55.5)	**0.01**
Triglycerides, mg/dL	152.0 (128.8–192.8)	148.0 (109.2–245.0)	0.74
D-dimer, ng/mL	271.5 (169.3–371.8)	217.0 (103.4–397.4)	0.33

*Note:* Bold *p* value corresponds to statistically significant differences.

^a^The median (IQR) is shown.

^b^Mann–Whitney test.

## Data Availability

The data that support the findings of this study are available from the corresponding author upon reasonable request.

## Supporting Information

Supporting Information Additional supporting information can be found online in the Supporting Information section. Table S1. Antibodies used to evaluate the phenotype of circulating T cells. Table S2. Antibodies used to evaluate the functional capacity of total and HIV-specific CD8+ T cells. Figure S1. Scatter plot showing the digital PCR results of an individual patient. Figure S2. Gating strategy to analyze the ex vivo phenotype of circulating T cells. Figure S3. Representative gating strategy to evaluate the frequency of HIV-specific T cells.
