# Supplementary material for: Benefits of INSTI-Based Regimens in a Real-World Setting of People Living With HIV-1 in Colombia
Source: Int J Microbiol. 2025 Sep 10;2025:9081023. doi: 10.1155/ijm/9081023 (PMC12443516; doi:10.1155/ijm/9081023)
Supplement: Supporting Information — Additional supporting information can be found online in the Supporting Information section. Table S1. Antibodies used to evaluate the phenotype of circulating T cells. Table S2. Antibodies used to evaluate the functional capacity of total and HIV-specific CD8+ T cells. Figure S1. Scatter plot showing the digital PCR results of an individual patient. Figure S2. Gating strategy to analyze the ex vivo phenotype of circulating T cells. Figure S3. Representative gating strategy to evaluate the frequency of HIV-specific T cells. [file 9081023.f1.docx]

**SUPPLEMENTAL DIGITAL CONTENT**

**Supplemental Table 1. Antibodies used to evaluate the phenotype of circulating T cells.**

| Antibody | Fluorochrome | Clone | Manufacturer | Catalog | Dose 1/ |
| --- | --- | --- | --- | --- | --- |
| CD3 | APC eFluor 780 | UCHT1 | Invitrogen | 47-0038-42 | 50 |
| CD4 | V450 | RPA-T4 | BD | 560345 | 80 |
| CD8 | PE Texas Red | 3B5 | Invitrogen | MHCD0817 | 100 |
| HLA-DR | PerCP Cy5.5 | G46-6 | BD | 552764 | 40 |
| CD38 | BV510 | HIT2 | BD | 563251 | 80 |
| CCR7 | PE Cy7 | 3D12 | BD | 560922 | 40 |
| CD45RA | Alexa Fluor 700 | HI100 | BD | 560673 | 167 |

**Supplemental Table 2. Antibodies used to evaluate the functional capacity of total and HIV-specific CD8^+^ T cells.**

| **Antibody** | **Clone** | **Manufacturer** | **Catalog** | **Dose 1/** |
| --- | --- | --- | --- | --- |
| CD3 Alexa Fluor 700 | UCHT1 | eBioscience | 56-0038-42 | 100 |
| CD4 APC efluor 780 | OKT4 | eBioscience | 47-0049-42 | 200 |
| CD8 efluor 450 | OKT8 | eBioscience | 48-0086-42 | 320 |
| CD69 ECD | TP1.55.3 | Beckman Coulter | 6607110 | 640 |
| CD137 (4-1BB) PE | 4B4-1 | Biolegend | 309804 | 320 |
| OX40 (CD134) PE Cy7 | ACT35 | Biolegend | 350012 | 1333 |

**Supplemental Figure 1.** Scatt Scatter plot showing the digital PCR results of an individual patient.


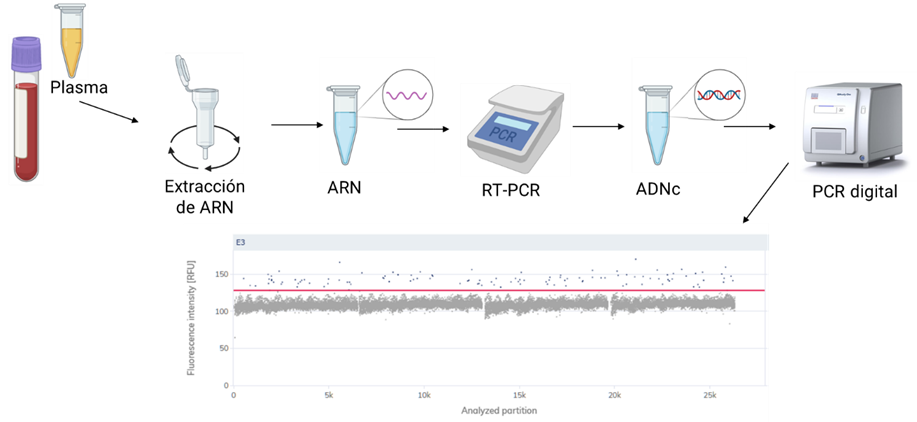


Each point represents one of the 26,000 PCR replicates; the X-axis displays the analyzed partitions (PCR reactions), while the Y-axis indicates fluorescence intensity (RFU). The red line marks the "threshold" above which a partition is considered positive.

**Supplemental Figure 2.** Gating strategy to analyze the ex vivo phenotype of circulating T cells.

**Supplemental Figure 3.** Representative gating strategy to evaluate the frequency of HIV-specific T cells.
